# Supplementary figures and images for: In silico analyses of penicillin binding proteins in Burkholderia pseudomallei uncovers SNPs with utility for phylogeography, species differentiation, and sequence typing
Source: PLoS Negl Trop Dis. 2022 Apr 13;16(4):e0009882. doi: 10.1371/journal.pntd.0009882 (PMC9037935; doi:10.1371/journal.pntd.0009882)

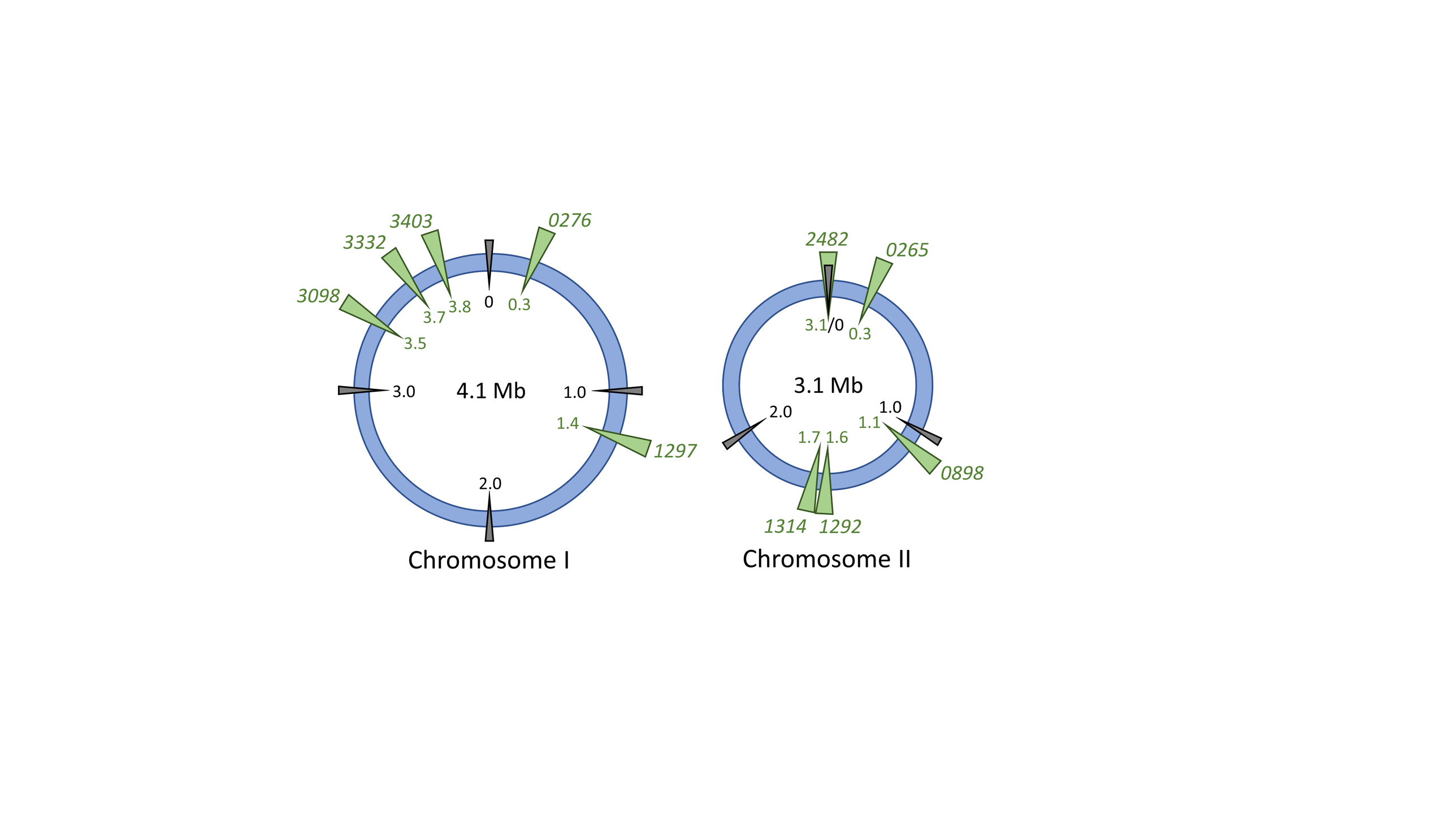

Supplement: S1 Fig — Loci tags are shown in green outside circular chromosomes. Loci positions within each chromosome are shown in green and have been rounded to the nearest decimal (Mb). (TIF) [file pntd.0009882.s001.tif]
