# Supplementary material for: In silico analyses of penicillin binding proteins in Burkholderia pseudomallei uncovers SNPs with utility for phylogeography, species differentiation, and sequence typing
Source: PLoS Negl Trop Dis. 2022 Apr 13;16(4):e0009882. doi: 10.1371/journal.pntd.0009882 (PMC9037935; doi:10.1371/journal.pntd.0009882)
Supplement: S1 Table — Information not available (--), Australia, Northern Territory (NT). (DOCX) [file pntd.0009882.s002.docx]

**S1 Table**. Initial set of *Burkholderia* isolates analyzed with the corresponding genome accession numbers used.

| ***B. pseudomallei* isolate** | **Origin /**  **Patient Travel or Residence** | **Isolation Source** | **Isolation Year** | **GenBank Accessions** |
| --- | --- | --- | --- | --- |
| 1026b (Reference) | Thailand | Human blood (septicemic) | 1993 | CP002833, CP002834 |
| K96243 | Thailand | Human | 1993 | CP009537, CP009538 |
| BGR | Thailand | -- | -- | CP008834, CP008835 |
| PHLS112 | Thailand | Human clinical | 1992 | CP009585, CP009586 |
| 1710b | Thailand | -- | -- | CP000124, CP000125 |
| 406e | Thailand | Human | 1998 | CP009297, CP009298 |
| 1106a | Thailand | Pus (liver abscess) | 1993 | CP008758, CP008759 |
| Mahidol-1106a | Thailand | -- | -- | CP008781, CP008782 |
| 576 | Thailand | Human | -- | CP008777, CP008778 |
| FDAARGOS 592 | Thailand | Clinical | -- | CP033705, CP033706 |
| FDAARGOS 593 | -- | -- | -- | CP033703, CP033704 |
| FDAARGOS 594 | Thailand | -- | -- | CP033701, CP033702 |
| HBPUB10134a | Thailand | Human tracheal Suction | 2010 | CP008911, CP008912 |
| 14M0960418 | Hong Kong | Blood Culture | 2014 | CP019042, CP019043 |
| BPHN1 | China | Goat | 2016 | CP023775, CP023776 |
| 350105 | Hainin, China | Water | 1976 | CP012093, CP012094 |
| BPC006 | Hainin, China | Blood from TypeII diabetes patient with abscesses | 2008 | CP003781, CP003782 |
| vgh16W | Taiwan | Human blood | 2001 | CP012517, CP012518 |
| vgh16R | Taiwan | Human blood | 2001 | CP012515, CP012516 |
| vgh07 | Taiwan | Human blood | 1996 | CP010973, CP010974 |
| Pasteur 52237 | Vietnam | -- | -- | CP009898, CP009899 |
| MS | Malaysia | Human | 2015 | CP016636, CP016637 |
| PMC2000 | Malaysia | Human | 2000 | CP025302, CP025303 |
| D286 | Malaysia | Clinical | 1986 | CP025306, CP025307 |
| H10 | Pahang, Malaysia | Human | 1995 | CP025300, CP25301 |
| R15 | Malaysia | Human | 2005 | CP025304, CP025305 |
| 982 | Pahang, Malaysia | Pus | 2015 | CP012576, CP012577 |
| M1 | Malaysia | Human | 2015 | CP016638, CP016639 |
| Strain 9 | Pakistan | -- | -- | CP008754, CP008755 |
| VB3253 | India | Human blood aspirate | 2019 | CP040531, CP040532 |
| VB2514 | India | Human blood aspirate | 2019 | CP040551, CP040552 |
| Bps 110 | Sri Lanka | Human blood | 2015 | CP036451, CP036452 |
| Bps 111 | Sri Lanka | Human blood | 2015 | CP036453, CP036454 |
| Bps 112 | Sri Lanka | Human blood | 2015 | CP037975, CP037976 |
| Bps 114 | Sri Lanka | Human blood | 2015 | CP037973, CP037974 |
| Bps 115 | Sri Lanka | Human blood | 2015 | CP037757, CP037758 |
| Bps 116 | Sri Lanka | Human blood | 2015 | CP037759, CP037760 |
| Bps 122 | Sri Lanka | Human blood | 2015 | CP038194, CP038195 |
| Bps 123 | Sri Lanka | Human joint fluid/blood | 2015 | CP037969, CP037970 |
| Bps 133 | Sri Lanka | Human blood | 2015 | CP037971, CP037972 |
| BSR | -- | -- | -- | CP009127, CP009128 |
| BGK | Thailand | -- | -- | CP008916, CP008917 |
| NCTC 13178 | Australia | Human brain (*post mortem*) | -- | CP004001, CP004002 |
| NCTC 13179 | Australia | Human skin ulcer | -- | CP003976, CP003977 |
| NAU35A-3 | Australia | Soil | 2006 | CP004377, CP004378 |
| NAU20B-16 | Australia | Soil | 2006 | CP004003, CP004004 |
| TSV 202 | Australia | Environmental | -- | CP009156, CP009157 |
| TSV 48 | Australia | Environmental | -- | CP009160, CP009161 |
| BDP | Australia, NT | Brain | 1994 | CP009209, CP009210 |
| MSHR146 | Australia | Right udder (goat) | 1992 | CP004042, CP004043 |
| MSHR62 | Australia | Human clinical | -- | CP009235, CP009234 |
| MSHR5858 | Australia | Human sputum | 2011 | CP008891, CP008892 |
| MSHR2243 | Australia | Human clinical | -- | CP009270, CP009269 |
| MSHR840 | Australia, Ipswitch | Human brain tissue | 1999 | CP009473, CP009474 |
| MSHR511 | Australia | Throat (goat) | 1997 | CP004023, CP004024 |
| MSHR668 | Australia | Human brain | 1995 | CP009545, CP009546 |
| MSHR6755 | Australia, NT | Blood | 2012 | CP017046, CP017047 |
| MSHR4083 | Australia, NT | Human | 2010 | CP017050, CP017051 |
| MSHR7929 | Australia, NT | Blood | 2013 | CP017044, CP017045 |
| MSHR520 | Australia | Blood | 1998 | CP004368, CP004369 |
| MSHR305 | Australia, NT | Brain sample (autopsy) | 1994 | CP006469, CP006470 |
| MSHR5864 | Australia, NT | Blood | 2011 | CP017048, CP017049 |
| MSHR3763 | Australia, NT | Human | 2010 | CP017052, CP017053 |
| MSHR2543 | Australia | -- | -- | CP009477, CP009478 |
| MSHR491 | Australia, NT | Community water supply storage tank | 1997 | CP009484, CP009485 |
| MSHR1153 | Australia | Clinical | -- | CP009271, CP009272 |
| MSHR1435 | Australia, NT | Environment | 2002 | CP025264, CP025265 |
| MSHR3965 | Australia | Environment | -- | CP009152, CP009153 |
| MSHR1655 | Australia | -- | -- | CP008779, CP008780 |
| Bp1651 | USA / Australia | Human sputum | -- | CP012041, CP012042 |
| Burk178-Type1 | Australia | Human sputum | 2011 | CP016909, CP016910 |
| Burk179-Type2 | Australia | Human sputum | 2011 | CP016911, CP016912 |
| K42 | Papua New Guinea | Environmental | -- | CP009162, CP00963 |
| B03 | Papua New Guinea | Environmental | -- | CP009150, CP009151 |
| A79A | Papua New Guinea | Environmental | -- | CP009165, CP009164 |
| VB976100 | Czech Republic | Pus of abscess (iguana) | 2014 | CP018054, CP018055 |
| PR1998 | Puerto Rico | Human clinical | 1998 | CP018369, CP018370 |
| PR1982 | Puerto Rico | Human clinical | 1982 | CP018367, CP018368 |
| PR2012 | Puerto Rico | Human clinical | 2012 | CP018393, CP018394 |
| PR2013a | Puerto Rico | Soil | 2013 | CP018406, CP018407 |
| PR2013b | Puerto Rico | Soil | 2103 | CP018408, CP018409 |
| FL2012 | FL, USA / Trinidad | Human clinical | 2012 | CP018391, CP018392 |
| MX2013 | CA, USA /  Mexico & Vietnam | Human clinical | 2013 | CP018395, CP018396, CP018397 |
| TX2004 | TX, USA / SE Asia | Human clinical | 2004 | CP018375, CP018376 |
| IL2014 | IL, USA / Mexico | Human clinical | 2014 | CP018414, CP018415 |
| VEN1976 | Venezuela / Unknown | Human clinical | 1976 | CP018371, CP018372 |
| 7894 | Ecuador / Unknown | Human clinical | 1962 | CP018373, CP018374 |
| CA2007 | CA, USA / Unknown | Human clinical | 2007 | CP018418, CP018419 |
| CA2009 | CA, USA / Mexico | Human clinical | 2009 | CP018380, CP018381 |
| PB1007001 | AZ, USA / Costa Rica | Human clinical | 2009 | CP018387, CP018388 |
| OH2013 | OH, USA / None | Human clinical | 2013 | CP018400, CP018401 |
| NY2010 | NY, USA / Aruba | Human clinical | 2010 | CP018384, CP018386 |
| Swiss2010 | Switzerland / Martinique | Human clinical | 2010 | CP018389, CP018390 |
| GA2015 | GA, USA / Panama & Peru | Human clinical | 2015 | CP018416, CP018417 |
| TX2015 | TX, USA / Mexico | Human clinical | 2015 | CP018412, CP018413 |
| CA2010 | CA, USA / Unknown | Human clinical | 2010 | CP018382, CP018383 |
| CA2013a | CA, USA / Unknown | Human clinical | 2013 | CP018398, CP018399 |
| MX2014 | CA, USA / Mexico | Human clinical | 2014 | CP018410, CP018411 |
| RI2013a | RI, USA / Guatemala | Human clinical | 2013 | CP018402, CP018403 |
| RI2013b | RI, USA / Guatemala | Human clinical | 2013 | CP018404, CP018405 |
| PB08298010 | AZ, USA / Unknown | Human clinical | 2008 | CP009550, CP009551 |
| ***B. mallei* isolate** | **Origin** | **Source** | **Year** | **Accession** |
| ATCC23344 | -- | -- | -- | CP000010, CP000011 |
| NCTC10247 | Turkey | -- | -- | CP007801, CP007802 |
| Bahrain1 | Bahrain | Horse | 2011 | CP017175, CP017176 |
| JHU | USA | Human | 2000 | CP009931, CP009932 |
| FMH | USA | Human | 2000 | CP009929, CP009930 |
| 2002721276 | -- | -- | 1956 | CP010065, CP010066 |
| 2002734306 | UK | -- | -- | CP009707, CP009708 |
| India86-567-2 | India | Mule |  | CP009642, CP009643 |
| strain 11 | Turkey | Human | 1949 | CP009587, CP009588 |
| strain 6 | -- | -- | -- | CP008710, CP008711 |
| 2002734299 | Hungary | Guinea Pig | 1961 | CP009337, CP009338 |
| BMQ | India | Horse |  | CP008722, CP008723 |
| NCTC10229 | -- | -- | -- | CP000545, CP000546 |
| SAVP1 | -- | -- | -- | CP000525, CP000526 |
| KC_1092 | Iran | -- | -- | CP009942, CP009943 |
| 2000031063 | -- | -- | -- | CP008731, CP008732 |
| Turkey10 | Turkey | -- | -- | CP010348, CP010349 |
| Turkey9 | Turkey | -- | -- | CP009741, CP009742 |
| Turkey8 | Turkey | -- | -- | CP009739, CP009740 |
| Turkey7 | Turkey | -- | -- | CP009737, CP009738 |
| Turkey6 | Turkey | -- | -- | CP009735, CP009736 |
| Turkey5 | Turkey | -- | -- | CP009733, CP009734 |
| Turkey4 | Turkey | -- | -- | CP009731, CP009732 |
| Turkey3 | Turkey | -- | -- | CP009729, CP009730 |
| Turkey2 | Turkey | -- | -- | CP009727, CP009728 |
| Turkey1 | Turkey | -- | -- | CP009725, CP009726 |
| ***B. thailandensis* isolate** | **Origin** | **Source** | **Year** | **Accession** |
| E444 | Thailand | Soil | 2002 | CP004117, CP004118 |
| E254 | Thailand | -- | 1992 | CP004381, CP004382 |
| E264 | Thailand | Rice Field Soil | -- | CP008785, CP008786 |
| H0587 | LA, USA | Human Pleural Wound | 1997 | CP004089 CP004090 |
| MSMB59 | Australia, NT | Soil | 2006 | CP013407 CP013408 |
| FDAARGOS 237 | USAMRIID | Water | 2009 | CP020390, CP020389 |
| FDAARGOS 238 | USAMRIID | Human Pleural Wound | 2009 | CP020392, CP020391 |
| FDAARGOS 241 | USAMRIID/CDC | -- | 2011 | CP022214, CP022215 |
| FDAARGOS 242 | Thailand | -- | 2011 | CP022217 CP022216 |
| FDAARGOS 426 | USAMRIID | Environment | -- | CP023499, CP023498 |
| MSMB121 | Australia | Soil | 2007 | CP004095, CP004096 |
| 20027211643 | -- | -- | 2002 | CP009601, CP009602 |
| 34 | -- | -- | 2002 | CP010017, CP010018 |
| USAMRU Malaysia #20 | Malaysia | -- | -- | CP004383, CP004384 |
| 2002721121 | USA | -- | -- | CP013409 CP013410 |
| 2003015869 | TX, USA | Human NAU subculture | 2003 | CP013360, CP013361 |
| 2002721723 | -- | Human | -- | CP004097, CP004098 |
