# Supplementary material for: In silico analyses of penicillin binding proteins in Burkholderia pseudomallei uncovers SNPs with utility for phylogeography, species differentiation, and sequence typing
Source: PLoS Negl Trop Dis. 2022 Apr 13;16(4):e0009882. doi: 10.1371/journal.pntd.0009882 (PMC9037935; doi:10.1371/journal.pntd.0009882)
Supplement: S3 Table — Nucleotides with phylogeographic utility are shown in black font and nucleotides used to differentiate Burkholderia species are shown in gray font. (DOCX) [file pntd.0009882.s004.docx]

**S3 Table**. Dual Locus Sequence Typing (DLST) results for the initial set of *B. pseudomallei* and *B. mallei* strains.

| ***B. pseudomallei* isolate** | **DLST** | **nucleotide position (*I0276*)** | | | | | **nucleotide position (*II1314)*** | | | | | |
| --- | --- | --- | --- | --- | --- | --- | --- | --- | --- | --- | --- | --- |
|  | **Sequence Type** | **141** | **268** | **888** | **1473** | **1629** | **243** | **265** | **273** | **575** | **703** | **854** |
| 1026b (Reference) | 1 | C | C | C | C | T | G | G | T | T | A | A |
| K96243 | 1 | C | C | C | C | T | G | G | T | T | A | A |
| BGR | 1 | C | C | C | C | T | G | G | T | T | A | A |
| PHLS112 | 5 | C | C | C | C | T | A | G | C | C | A | C |
| 1710b | 1 | C | C | C | C | T | G | G | T | T | A | A |
| 406e | 13 | C | C | C | C | T | G | G | C | C | A | A |
| 1106a | 4 | C | C | C | C | T | G | G | C | T | A | A |
| Mahidol-1106a | 4 | C | C | C | C | T | G | G | C | T | A | A |
| 576 | 3 | C | C | C | C | T | G | G | C | C | A | C |
| FDAARGOS 592 | 3 | C | C | C | C | T | G | G | C | C | A | C |
| FDAARGOS 593 | 5 | C | C | C | C | T | A | G | C | C | A | C |
| FDAARGOS 594 | 3 | C | C | C | C | T | G | G | C | C | A | C |
| HBPUB10134a | 1 | C | C | C | C | T | G | G | T | T | A | A |
| 14M0960418 | 4 | C | C | C | C | T | G | G | C | T | A | A |
| BPHN1 | 13 | C | C | C | C | T | G | G | C | C | A | A |
| 350105 | 24 | C | C | C | C | T | G | G | C | C | G | A |
| BPC006 | 4 | C | C | C | C | T | G | G | C | T | A | A |
| vgh16W | 1 | C | C | C | C | T | G | G | T | T | A | A |
| vgh16R | 1 | C | C | C | C | T | G | G | T | T | A | A |
| vgh07 | 1 | C | C | C | C | T | G | G | T | T | A | A |
| Pasteur 52237 | 3 | C | C | C | C | T | G | G | C | C | A | C |
| MS | 9 | C | T | C | C | T | G | G | C | C | G | C |
| PMC2000 | 4 | C | C | C | C | T | G | G | C | T | A | A |
| D286 | 4 | C | C | C | C | T | G | G | C | T | A | A |
| H10 | 1 | C | C | C | C | T | G | G | T | T | A | A |
| R15 | 4 | C | C | C | C | T | G | G | C | T | A | A |
| 982 | 1 | C | C | C | C | T | G | G | T | T | A | A |
| M1 | 9 | C | T | C | C | T | G | G | C | C | G | C |
| Strain 9 | 3 | C | C | C | C | T | G | G | C | C | A | C |
| VB3253 | 6 | C | C | C | C | T | A | G | C | C | G | C |
| VB2514 | 6 | C | C | C | C | T | A | G | C | C | G | C |
| Bps 110 | 2 | C | C | C | C | T | G | G | C | C | G | C |
| Bps 111 | 2 | C | C | C | C | T | G | G | C | C | G | C |
| Bps 112 | 6 | C | C | C | C | T | A | G | C | C | G | C |
| Bps 114 | 6 | C | C | C | C | T | A | G | C | C | G | C |
| Bps 115 | 6 | C | C | C | C | T | A | G | C | C | G | C |
| Bps 116 | 6 | C | C | C | C | T | A | G | C | C | G | C |
| Bps 122 | 6 | C | C | C | C | T | A | G | C | C | G | C |
| Bps 123 | 2 | C | C | C | C | T | G | G | C | C | G | C |
| Bps 133 | 6 | C | C | C | C | T | A | G | C | C | G | C |
| BSR | 13 | C | C | C | C | T | G | G | C | C | A | A |
| BGK | 1 | C | C | C | C | T | G | G | T | T | A | A |
| NCTC 13178 | 2 | C | C | C | C | T | G | G | C | C | G | C |
| NCTC 13179 | 2 | C | C | C | C | T | G | G | C | C | G | C |
| NAU35A-3 | 7 | C | C | C | C | T | G | G | C | T | G | C |
| NAU20B-16 | 2 | C | C | C | C | T | G | G | C | C | G | C |
| TSV 202 | 3 | C | C | C | C | T | G | G | C | C | A | C |
| TSV 48 | 2 | C | C | C | C | T | G | G | C | C | G | C |
| BDP | 7 | C | C | C | C | T | G | G | C | T | G | C |
| MSHR146 | 2 | C | C | C | C | T | G | G | C | C | G | C |
| MSHR62 | 2 | C | C | C | C | T | G | G | C | C | G | C |
| MSHR5858 | 24 | C | C | C | C | T | G | G | C | C | G | A |
| MSHR2243 | 2 | C | C | C | C | T | G | G | C | C | G | C |
| MSHR840 | 3 | C | C | C | C | T | G | G | C | C | A | C |
| MSHR511 | 2 | C | C | C | C | T | G | G | C | C | G | C |
| MSHR668 | 2 | C | C | C | C | T | G | G | C | C | G | C |
| MSHR6755 | 2 | C | C | C | C | T | G | G | C | C | G | C |
| MSHR4083 | 7 | C | C | C | C | T | G | G | C | T | G | C |
| MSHR7929 | 2 | C | C | C | C | T | G | G | C | C | G | C |
| MSHR520 | 7 | C | C | C | C | T | G | G | C | T | G | C |
| MSHR305 | 7 | C | C | C | C | T | G | G | C | T | G | C |
| MSHR5864 | 2 | C | C | C | C | T | G | G | C | C | G | C |
| MSHR3763 | 7 | C | C | C | C | T | G | G | C | T | G | C |
| MSHR2543 | 9 | C | T | C | C | T | G | G | C | C | G | C |
| MSHR491 | 2 | C | C | C | C | T | G | G | C | C | G | C |
| MSHR1153 | 2 | C | C | C | C | T | G | G | C | C | G | C |
| MSHR1435 | 2 | C | C | C | C | T | G | G | C | C | G | C |
| MSHR3965 | 2 | C | C | C | C | T | G | G | C | C | G | C |
| MSHR1655 | 2 | C | C | C | C | T | G | G | C | C | G | C |
| Bp1651 | 2 | C | C | C | C | T | G | G | C | C | G | C |
| Burk178-Type1 | 7 | C | C | C | C | T | G | G | C | T | G | C |
| Burk179-Type2 | 7 | C | C | C | C | T | G | G | C | T | G | C |
| K42 | 18 | C | C | C | T | T | G | G | C | C | G | C |
| B03 | 18 | C | C | C | T | T | G | G | C | C | G | C |
| A79A | 18 | C | C | C | T | T | G | G | C | C | G | C |
| VB976100 | 17 | T | C | C | C | T | G | G | C | T | A | A |
| PR1998 | 12 | C | T | C | C | T | G | T | T | T | A | A |
| PR1982 | 12 | C | T | C | C | T | G | T | T | T | A | A |
| PR2012 | 12 | C | T | C | C | T | G | T | T | T | A | A |
| PR2013a | 12 | C | T | C | C | T | G | T | T | T | A | A |
| PR2013b | 12 | C | T | C | C | T | G | T | T | T | A | A |
| FL2012 | 12 | C | T | C | C | T | G | T | T | T | A | A |
| MX2013 | 1 | C | C | C | C | T | G | G | T | T | A | A |
| TX2004 | 10 | C | T | C | C | T | G | G | T | T | A | A |
| IL2014 | 10 | C | T | C | C | T | G | G | T | T | A | A |
| VEN1976 | 10 | C | T | C | C | T | G | G | T | T | A | A |
| 7894 | 10 | C | T | C | C | T | G | G | T | T | A | A |
| CA2007 | 25 | C | T | C | C | T | G | G | T | C | A | A |
| CA2009 | 11 | C | T | C | C | T | G | G | C | C | A | A |
| PB1007001 | 10 | C | T | C | C | T | G | G | T | T | A | A |
| OH2013 | 3 | C | C | C | C | T | G | G | C | C | A | C |
| NY2010 | 10 | C | T | C | C | T | G | G | T | T | A | A |
| Swiss2010 | 10 | C | T | C | C | T | G | G | T | T | A | A |
| GA2015 | 17 | T | C | C | C | T | G | G | C | T | A | A |
| TX2015 | 22 | T | C | C | C | T | G | G | C | C | A | A |
| CA2010 | 5 | C | C | C | C | T | A | G | C | C | A | C |
| CA2013a | 25 | C | T | C | C | T | G | G | T | C | A | A |
| MX2014 | 17 | T | C | C | C | T | G | G | C | T | A | A |
| RI2013a | 17 | T | C | C | C | T | G | G | C | T | A | A |
| RI2013b | 17 | T | C | C | C | T | G | G | C | T | A | A |
| PB08298010 | 3 | C | C | C | C | T | G | G | C | C | A | C |
| ***B. mallei* isolate** | **DLST** | **nucleotide position (*I0276*)** | | | | | **nucleotide position (*II1314)*** | | | | | |
|  | **Sequence Type** | **141** | **268** | **888** | **1473** | **1629** | **243** | **265** | **273** | **575** | **703** | **854** |
| ATCC23344 | 32 | C | T | T | C | C | A | G | C | C | G | C |
| NCTC10247 | 32 | C | T | T | C | C | A | G | C | C | G | C |
| Bahrain1 | 32 | C | T | T | C | C | A | G | C | C | G | C |
| JHU | 32 | C | T | T | C | C | A | G | C | C | G | C |
| FMH | 32 | C | T | T | C | C | A | G | C | C | G | C |
| 2002721276 | 32 | C | T | T | C | C | A | G | C | C | G | C |
| 2002734306 | 32 | C | T | T | C | C | A | G | C | C | G | C |
| India86-567-2 | 32 | C | T | T | C | C | A | G | C | C | G | C |
| strain 11 | 32 | C | T | T | C | C | A | G | C | C | G | C |
| strain 6 | 32 | C | T | T | C | C | A | G | C | C | G | C |
| 2002734299 | 32 | C | T | T | C | C | A | G | C | C | G | C |
| BMQ | 32 | C | T | T | C | C | A | G | C | C | G | C |
| NCTC10229 | 32 | C | T | T | C | C | A | G | C | C | G | C |
| SAVP1 | 32 | C | T | T | C | C | A | G | C | C | G | C |
| KC_1092 | 32 | C | T | T | C | C | A | G | C | C | G | C |
| 2000031063 | 32 | C | T | T | C | C | A | G | C | C | G | C |
| Turkey10 | 32 | C | T | T | C | C | A | G | C | C | G | C |
| Turkey9 | 32 | C | T | T | C | C | A | G | C | C | G | C |
| Turkey8 | 32 | C | T | T | C | C | A | G | C | C | G | C |
| Turkey7 | 32 | C | T | T | C | C | A | G | C | C | G | C |
| Turkey6 | 32 | C | T | T | C | C | A | G | C | C | G | C |
| Turkey5 | 32 | C | T | T | C | C | A | G | C | C | G | C |
| Turkey4 | 32 | C | T | T | C | C | A | G | C | C | G | C |
| Turkey3 | 32 | C | T | T | C | C | A | G | C | C | G | C |
| Turkey2 | 32 | C | T | T | C | C | A | G | C | C | G | C |
| Turkey1 | 32 | C | T | T | C | C | A | G | C | C | G | C |
