# Supplementary material for: In silico analyses of penicillin binding proteins in Burkholderia pseudomallei uncovers SNPs with utility for phylogeography, species differentiation, and sequence typing
Source: PLoS Negl Trop Dis. 2022 Apr 13;16(4):e0009882. doi: 10.1371/journal.pntd.0009882 (PMC9037935; doi:10.1371/journal.pntd.0009882)
Supplement: S4 Table — 99.7% (1,442/1,446) B. pseudomallei strains exhibited a minimum of ≥98.8% identity to II1314 encoding the PBP-3 (3) homolog in B. pseudomallei 1026b. (*) The remaining four strains (3001161869, BP-6260, BP-6887, RNS8-BP1) demonstrated poor alignment and low sequence identity to II1314, accounting for the lower minimum percent identity of 67.7%. These strains were excluded from DLST analysis. (DOCX) [file pntd.0009882.s005.docx]

**S4 Table**. Alignment results for 10 genes encoding putative PBPs in *B. pseudomallei* 1026b with 1,446 *B. pseudomallei* RefSeq genomes.

|  | **Percent Identity** | | | | **Bit-score** | | | |
| --- | --- | --- | --- | --- | --- | --- | --- | --- |
| Locus (1026b) | Min | Max | Mean | St. Dev | Min | Max | Mean | St. Dev |
| ***I0276*** | 99.7 | 100 | 99.9 | 0.1 | 3298 | 3323 | 3316.4 | 6.0 |
| ***II1292*** | 98.6 | 100 | 99.7 | 0.2 | 2059 | 3214 | 3186.0 | 34.4 |
| ***II1314*** | 67.7* | 100 | 99.6 | 1.2 | 401 | 3047 | 3017.3 | 110.0 |
| ***I3332*** | 97.4 | 100 | 99.5 | 0.4 | 3345 | 4345 | 4297.5 | 38.0 |
| ***II0898*** | 67.6 | 100 | 98.4 | 1.2 | 88 | 4908 | 4625.0 | 259.7 |
| ***I3403*** | 95.7 | 100 | 99.8 | 0.1 | 2777 | 4318 | 4292.6 | 63.6 |
| ***I1297*** | 77.5 | 100 | 99.8 | 0.7 | 1644 | 4551 | 4525.5 | 123.0 |
| ***II0265*** | 99.4 | 100 | 99.8 | 0.1 | 1966 | 3864 | 3839.3 | 83.6 |
| ***II2482*** | 97.7 | 100 | 99.8 | 0.1 | 4094 | 4648 | 4620.2 | 38.0 |
| ***I3098*** | 67.8 | 100 | 99.8 | 0.9 | 165 | 2370 | 2356.9 | 59.7 |
