# Supplementary material for: In silico analyses of penicillin binding proteins in Burkholderia pseudomallei uncovers SNPs with utility for phylogeography, species differentiation, and sequence typing
Source: PLoS Negl Trop Dis. 2022 Apr 13;16(4):e0009882. doi: 10.1371/journal.pntd.0009882 (PMC9037935; doi:10.1371/journal.pntd.0009882)
Supplement: S5 Table — All RefSeq genomes are publicly available (NCBI) and geographic information was acquired from the BioSample database. DLSTs were assigned in numerical order based on the number of strains in each ST, highest to lowest. (+) UK laboratory cultures of B. pseudomallei K96243, originally from Thailand [60]. (*) ITS Type C. (#) 1 of 2 strains is Bp1651, Australian in origin. (DOCX) [file pntd.0009882.s006.docx]

**S5 Table**. DLST results and geographic origin for the 1,523 *B. pseudomallei* and *B. mallei* RefSeq genomes.

| **ST** | **DLST Sequence** | **Total # of Genomes** | **Country (# of strains)** |
| --- | --- | --- | --- |
| 1 | CCCCTGGTTAA | 451 | Thailand (416), Singapore (11), Malaysia (8), Vietnam (5), UK (4)^+^, Taiwan (3), Bangladesh (1), China (1), New Zealand (1), USA (1) |
| 2 | CCCCTGGCCGC | 220 | Australia (110), Thailand (100), Malaysia (5), Sri Lanka (3), USA (2)^#^ |
| 3 | CCCCTGGCCAC | 205 | Thailand (105), Singapore (48), Australia (30), Malaysia (7), France (5), Pakistan (3), Bangladesh (2), Papua New Guinea (1), Portugal (1), UK (1), USA (1), Vietnam (1) |
| 4 | CCCCTGGCTAA | 157 | Thailand (122), Singapore (26), Malaysia (5), China (1), Hong Kong (1), South Korea (1), Vietnam (1) |
| 5 | CCCCTAGCCAC | 92 | Thailand (91), USA (1)* |
| 6 | CCCCTAGCCGC | 74 | Thailand (66), Sri Lanka (6), Israel (1), Vietnam (1) |
| 7 | CCCCTGGCTGC | 67 | Australia (63), Thailand (4) |
| 8 | CTCCTAGCCAC | 34 | Thailand (34) |
| 9 | CTCCTGGCCGC | 32 | Australia (23), Malaysia (8), Thailand (1) |
| 10 | CTCCTGGTTAA | 13 | USA (9), Ecuador (2), Switzerland (1) Venezuela (1) |
| 11 | CTCCTGGCCAA | 13 | Australia (12), USA (1) |
| 12 | CTCCTGTTTAA | 11 | USA (6), Puerto Rico (5) |
| 13 | CCCCTGGCCAA | 10 | Thailand (7), Australia (2), China (1) |
| 14 | CTCCTGGCCAC | 8 | Australia (6), New Zealand (1), Thailand (1) |
| 15 | CCCCTGGTTAC | 7 | Thailand (7) |
| 16 | CCCCTAGCCAA | 6 | Thailand (6) |
| 17 | TCCCTGGCTAA | 5 | USA (4), Czech Republic (1) |
| 18 | CCCTTGGCCGC | 5 | Papua New Guinea (5) |
| 19 | CCCCTGGTCAC | 5 | Thailand (5) |
| 20 | CCCCTGGCTAC | 5 | Australia (4), Thailand (1) |
| 21 | CCCCTAGCTGC | 4 | Thailand (4) |
| 22 | TCCCTGGCCAA | 3 | Mexico (2), USA (1) |
| 23 | CCCCTGGTCAA | 3 | Thailand (3) |
| 24 | CCCCTGGCCGA | 3 | Australia (3) |
| 25 | CTCCTGGTCAA | 2 | USA (2) |
| 26 | CCCCTGGTTGC | 2 | Thailand (2) |
| 27 | CTCCTGGCTGC | 1 | Australia (1) |
| 28 | CCCCTGGTCGC | 1 | Thailand (1) |
| 29 | CCCCTGGACGC | 1 | Australia (1) |
| 30 | CCCCTGGACAC | 1 | Australia (1) |
| 31 | CCCCTAGCCGA | 1 | Thailand (1) |
| 32 | CTTCCAGCCGC  (*B. mallei*) | 81 | 55/81 strains have associated geographic information:  Turkey (24), India (8), USA (6), Hungary (5), China (4), Myanmar (2), UK (2), Bahrain (1), Iran (1), Pakistan (1), Russia (1) |
